# Different Physiological Responses to Continuous Drought between Seedlings and Younger Individuals of *Haloxylon ammodendron*

**DOI:** 10.3390/plants12213683

**Published:** 2023-10-25

**Authors:** Lidan Chen, Minqing Li, Congjuan Li, Weihua Zheng, Ran Liu

**Affiliations:** 1 State Key Laboratory of Desert and Oasis Ecology, Xinjiang Institute of Ecology and Geography, Chinese Academy of Sciences, Urumqi 830011, China; lidanchen0228@163.com (L.C.); liminqing11@126.com (M.L.); 2 University of Chinese Academy of Sciences, Beijing 100049, China; 3 Fukang National Station of Observation and Research for Desert Ecosystem, Fukang 831505, China; 4 National Engineering Technology Research Center for Desert-Oasis Ecological Construction, Xinjiang Institute of Ecology and Geography, Chinese Academy of Sciences, Urumqi 830011, China; licj@ms.xjb.ac.cn; 5 Institute of Agricultural Quality Standards and Testing Technology, Xinjiang Academy of Agricultural Sciecnes, Urumuqi 830091, China; bachuzwh@163.com

**Keywords:** physiological response, net photosynthetic rate, stomatal conductance, potential maximum photochemical rate, non-structural carbohydrates

## Abstract

Drought is an important environmental factor that influences physiological processes in plants; however, few studies have examined the physiological mechanisms underlying plants’ responses to continuous drought. In this study, the seedlings and younger individuals of *Haloxylon ammodendron* were experimentally planted in the southern part of the Gurbantunggut Desert. We measured their photosynthetic traits, functional traits and non-structural carbohydrate contents (NSCs) in order to assess the effects of continuous drought (at 15-day and 30-day drought points) on the plants’ physiological responses. The results showed that at the 15-day (15 d) drought point, the leaf light-saturated net photosynthetic rate (A_n_) values of both the seedlings and the younger individuals were decreased (by −68.9% and −45.2%, respectively). The intrinsic water use efficiency (iWUE) of the seedlings was significantly lower than that of the control group (−52.2%), but there was no diffenrence of iWUE observed in younger individuals. At the 30-day (30 d) drought point, a decrease in the A_n_ (−129.8%) of the seedlings was induced via biochemical inhibition, with a lower potential maximum photochemical rate (Fv/Fm, 0.42) compared with the control group, while a decrease in the A_n_ (−52.3%) of the younger individuals was induced due to lower stomatal conductance (g_s_, −50.5%). Our results indicated that prolonged drought induced a greater risk of seedling mortality as the relatively limited ability of stomatal regulation may increase the possibility of massive embolism, resulting in hydraulic failure.

## 1. Introduction

The high and unexpected frequency of extreme climatic events and the effects of human activities have strongly affected the amount of water available for the growth of vegetation [1,2,3,4,5]. Drought is the principal limiting factor for plant physiological processes and growth in a natural ecosystem [6,7,8]. Vegetation in the desert is strongly constrained by low soil water availability induced by frequent global climatic changes and increasing human activities [9,10]. In particular, limited water availability can strongly reduce plant growth and even affect the survival of plant seedlings [11]. Plants in deserts are extremely sensitive to environmental changes and highly vulnerable to extreme drought events [12,13], and they exhibit markedly variable physiological and morphological strategies for withstanding drought [14]. Many studies have reported the different adaptation strategies of species [15,16,17]; however, few studies have investigated the different physiological response strategies of plants at different developmental stages to drought.

Plant response strategies to drought are complex and differ among ages and species, thereby influencing plant survival after drought events [4,18,19]. Many previous studies reported the eco-physiological responses of plants to drought stress, including morphological traits (increasing the leaf area per mass and reducing the leaf size, stem length and leaf length/width) and physiological traits (reducing net photosynthetic rates, stomatal conductance and leaf water potential) [11,20]. In addition, a plant’s physiological response to drought is markedly related to its mortality risk, species composition and ecosystem functions [15,21,22]. The leaf gas exchange, moreover, is an essential process in an ecosystem’s carbon cycle and also an important physiological trait which can be easily impacted by environmental factors [23,24,25]. A large number of studies have reported that the leaf light-saturated net photosynthetic rate (A_n_) decreases with decreasing water availability due to stomatal closure to save water and maintain hydraulic structural integrity [16,26]. With long-term drought, biochemical limitations to photosynthesis are generated via severe injury in biochemical processes, which further reduce the A_n_ value [27]. Additionally, a plant’s uptake and transportation of water are closely related to its acquisition of nutrients from soil [28]. Thus, low soil water availability could inhibit plant nutrient acquisition, which would further impair photosynthetic biochemical processes [29]. Aside from stomatal regulation, leaf desiccation and canopy wilt are two common means of reducing the transpiration area which affect carbon fixation as well [9]. Furthermore, positive stomatal regulation can reduce water loss but also limits CO_2_ availability, while negative stomatal regulation can maintain high CO_2_ availability but may lead to higher risks of stem cavitation and hydraulic failure [30,31]. According to the stomatal response to drought stress, the water use strategies of species can be divided into risky and conservative strategies [32]. Stomatal regulation strategies can reflect the plant water use theory that stomatal optimization can be framed in terms of maximizing marginal carbon gain through the intrinsic water use efficiency (iWUE) [33]. Water use strategies are closely related to living environmental factors [34,35]; for instance, Wright et al. [36] reported that dry-site species have a conservative strategy that leads to a lower stomatal conductance at a given photosynthetic rate. According to different stomatal regulating strategies, there are two hypotheses to explain the risk of mortality induced by drought: the risk of hydraulic failure related to low hydraulic status with negative stomatal regulation and the risk of carbon starvation induced by long-term low photosynthetic rate with positive stomatal regulation [21,37,38]. Furthermore, stomatal closure can maintain a relatively high midday leaf water potential to avoid or reduce embolism and subsequent hydraulic failure [39]. 

Regarding water potential, there is increasing recognition that it is an inherent hydraulic trait which can be strongly influenced not only by environment factors but also by a plant’s developmental stages [6]. Additionally, the leaf water potential of trees decreases with declining soil moisture and clearly affects the gas exchange process [20,40]. A low leaf water potential can maintain normal water uptake with little soil water availability to support leaf transpiration but also would increase the risk of xylem embolism and decrease hydraulic conductance [34]. A water potential that is too low also represents low levels of water transportation in stems and further influences biomass allocation and metabolism in organs [41]. Moreover, a leaf water potential below a critical threshold may result in hydraulic failure that can lead to plant mortality [42]. Additionally, leaf water potential is a key regulator of transpiration and a critical predictor of plant water status and mortality [19]; therefore, plants must regulate their water potential to maintain the integrity of the hydraulic net along the soil–plant–atmosphere continuum [34].

Apart from water potential, the response of non-structural carbohydrates (NSCs) content is a critical indicator of plant physiological status and can reflect the dual risks of hydraulic failure and carbon starvation [43]. NSC content in tree tissues is considered to be a measure of carbon supply for growth and a foundation of stress resistance [39,44]. An unexpected change in the NSC pools may lead to an unbalance between carbon demand and concurrent supply which may induce carbon starvation [45]. Indeed, NSCs serve multiple roles in plant tissues including in growth and metabolism [46,47], and also signaling, osmotic regulation and defense [48]. As reported by David et al. [49], the growth of trees is strongly associated with NSC storage when constrained by photosynthesis. In addition, NSC plays a crucial role in phloem transportation when refilling embolisms, which always occur under limited soil water availability conditions [44,50,51]. Starch and soluble sugars are considered as the main fractions of NSC [52]. However, they show different functions in plant tissues. For instance, starch plays a role in storage, defined as the resources that can be used in the future for growth or embolism repair after extreme drought stress in the xylem [43]; in contrast to starch, soluble sugars are important to osmotic regulation to maintain stable hydraulic conductance under drought stress [47]. The reduction in NSCs also reduces the biosynthesis of photosynthase, which could further inhibit photosynthesis [53]. A recent meta-analysis showed that starch and soluble sugars were differentially altered in organs as drought time increased and implied the possibility of NSC depletion, which may cause carbon starvation [54]. Changes in NSCs are variable among species and complex with different influencing factors, such as drought, heat and pests [44]. However, few studies have evaluated NSCs’ variable responses over extended drought duration with seedlings at different developmental stages. 

Many studies have reported plant physiological responses to drought stress, whereas few studies have compared the different continuous drought stress effects on seedlings at different developmental stages. *Haloxylon ammodendron* is a dominant species in arid regions in Central Asia and the main vegetation used for restoration in desert areas, such as the Gurbantunggut Desert [55]. *H. ammodendron* acclimates to drought mainly with a safe strategy dominated by positive stomatal closure and morphological regulation, including thinner and shorter leaves, suggesting it could be used in the sustainable development of ecological restoration areas [56]. Additionally, *H. ammodendron* is characterized by well-developed vertical roots of more than 10 m caused by its strong dependence on groundwater [57,58]. *H. ammodendron* seedlings and younger individuals are the two main objects for restoring vegetation [59]. Previous studies have investigated the eco-physiological adaption of *H. ammodendron* to drought in situ [56], but there is limited information about the impact of the developmental stage on *H. ammodendron* seedlings’ responses to drought stress. In this study, we compared seedlings and younger individuals at two different developmental stages of *H. ammodendron* to investigate whether the developmental stage impacts the physiological responses to extreme continuous drought. We hypothesized that: 1. There would be different limited factors of leaf photosynthesis during different drought periods, 2. Younger individuals respond to continuous drought stress better than seedlings due to a more stable physiological regulation ability.

## 2. Results

### 2.1. Leaf Photosynthetic Traits

Both drought treatment and the developmental stage had significant effects on An and R. While drought treatment alone did not significantly affect g_s_, its interaction with the developmental stage did (*p* = 0.015, Table 1). In addition, the developmental stage and its interaction with treatment duration had significant effects on A_n_ and R, especially on R values (*p* < 0.001, Table 1). 

**Figure 1:**
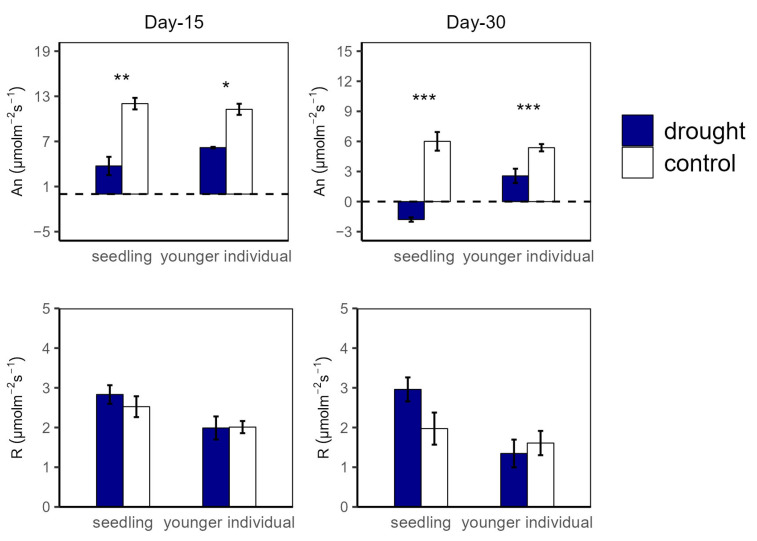
Net photosynthesis rate (A_n_) and dark respiration rate (R) of seedlings and younger individuals at the 15 d drought point (**left**) and the 30 d drought point (**right**). Bars indicate standard errors *(n* = 5). *, ** and *** represent significant difference at *p* < 0.05, *p* < 0.01 and *p* < 0.001, respectively. The dashed lines indicate the zero tick mark.

**Figure 2:**
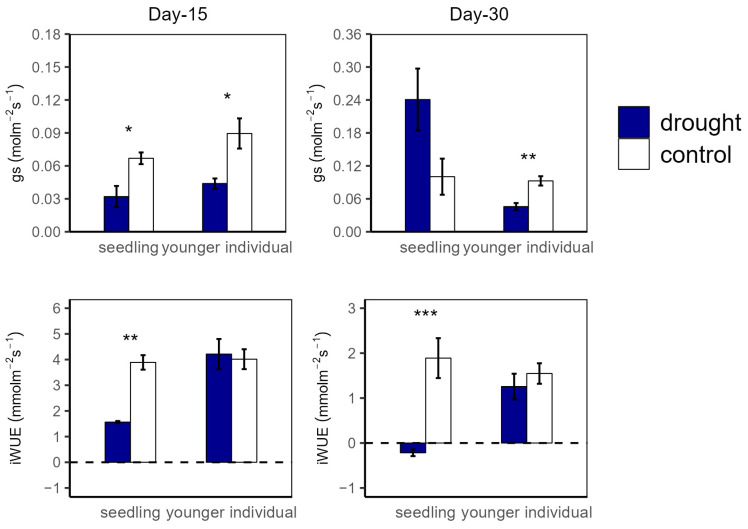
Stomatal conductance (gs) and intrinsic water use efficiency (iWUE) of seedlings and younger individuals at 15 d drought point (**left**) and 30 d drought point (**right**). Bars indicate standard errors (*n* = 5). *, ** and *** represent significant difference at *p* < 0.05, *p* < 0.01 and *p* < 0.001, respectively. The dashed lines indicate the zero tick mark.

At the 15 d drought point, A_n_ values were significantly higher in the control group compared to in the drought group for both seedlings (12.04 vs. 3.74 μmol m^−2^ s^−1^ and 11.27 vs. 6.18 μmol m^−2^ s^−1^ for seedlings and younger individuals, respectively), with no significant difference between the two developmental stages. For R, there was no significant difference between all groups (Figure 1). Additionally, both for seedlings and younger individuals under drought stress, g_s_ values were significantly lower than for the control group (*p* < 0.05, −52.2% and −50.5%, respectively) (Figure 2). 

At the 30 d drought point, the drought groups still had lower A_n_ values than the control groups; especially for the seedlings, the total photosynthetic rate was lower than the dark respiration rate (A_n_ < 0 μmol m^−2^ s^−1^) (Figure 1). Moreover, younger individuals had a lower g_s_ (0.05 μmol m^−2^ s^−1^) than the control group (0.09 μmol m^−2^ s^−1^), and seedlings showed a much higher g_s_ average value (0.24 μmol m^−2^ s^−1^) and a large standard deviation (SD = 0.13) (Figure 2).

**Figure 3:**
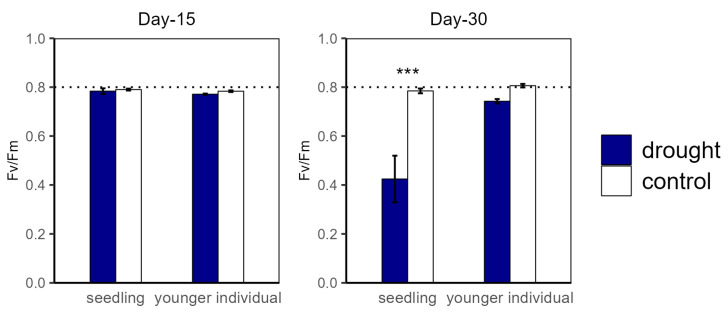
Potential maximum biochemical rate of PS II (Fv/Fm) of seedlings and younger individuals at 15 d drought point (**left**) and 30 d drought point (**right**). Bars indicate standard errors *(n* = 5). *** represents significant difference *p* < 0.001. The dashed lines indicate Fv/Fm = 0.8.

For the drought groups, the iWUE of seedlings was significantly lower than for the control groups, registering values of 1.57 vs. 4.21 μmol m^−2^ s^−1^ and −0.22 vs. 1.26 μmol m^−2^ s^−1^ at the 15 d and 30 d drought points, respectively. However, younger individuals showed no significant difference between the control and drought groups (Figure 2). Additionally, there was no significant difference in Fv/Fm at the 15 d drought point; nevertheless, the Fv/Fm (0.42) of seedlings was significantly lower compared with that of the control group at the 30 d drought point (Figure 3).

### 2.2. Plant Water Status

**Figure 4:**
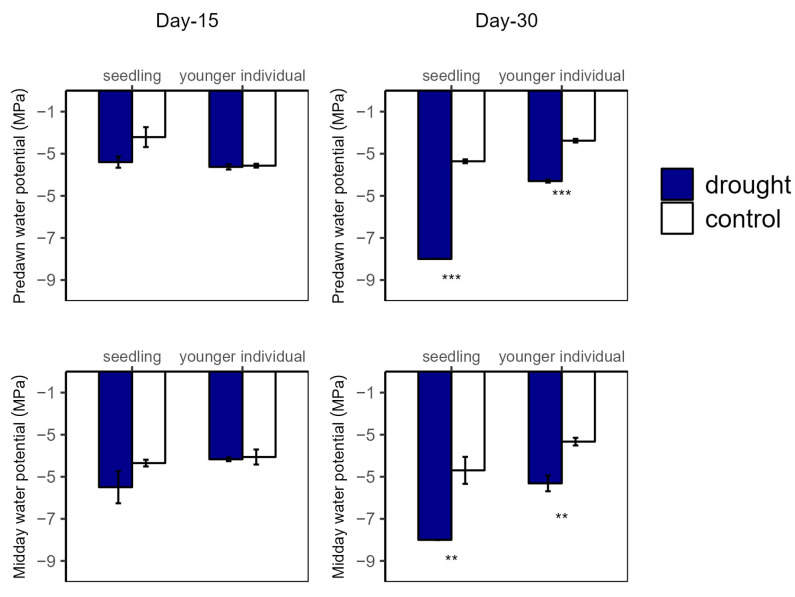
Predawn (**left**) and midday (**right**) leaf water potential of seedlings and younger individuals at 15 d drought point (**left**) and 30 d drought point (**right**). Bars indicate standard errors *(n* = 5). ** and *** represent significant difference at *p* < 0.01 and *p* < 0.001, respectively.

**Figure 5:**
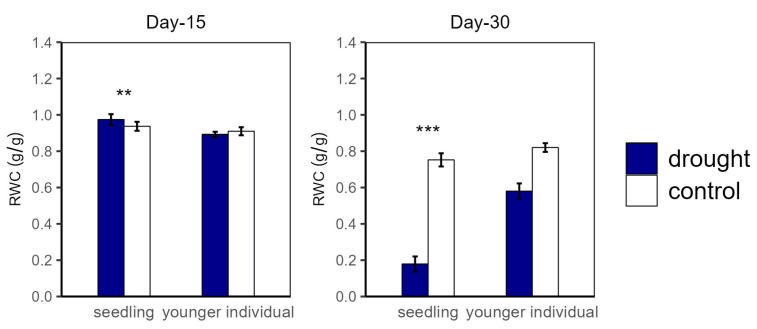
Relative water content (RWC) of seedlings and younger individuals at 15 d drought point (**left**) and 30 d drought point (**right**). Bars indicate standard errors (*n* = 5). ** and *** represent significant difference at *p* < 0.01 and *p* < 0.001, respectively.

Over the drought duration, all of the groups showed a similar tendency (Figure 4 and Figure 5). As shown in Figure 4 and Figure 5, for both seedlings and younger individuals at the 15 d drought point, there was no significant difference in leaf water potential and RWC between control and drought groups. It should be noted that, at the 30 d drought point, the seedlings’ leaf water potential in the drought group was lower than −8 MPa, which exceeded our scaling range; however, that of the control group (average values for Ψ_PD_ and Ψ_MD_ were −3.35 MPa and −4.70 MPa, respectively) was still significantly higher than −8 MPa. Therefore, we confirmed that leaf water potential of drought groups was lower than that of the control groups (*p* < 0.05) (Figure 4). In addition, the RWC of drought groups was significantly lower than that of control groups at the 30 d drought point, with values of 0.58 vs. 0.82 g·g^−1^ (−29.27%) and 0.18 vs. 0.75 g·g^−1^ (−79.17%) for younger individuals and seedlings, respectively (Figure 5).

### 2.3. Non-Structural Carbohydrate

**Figure 6:**
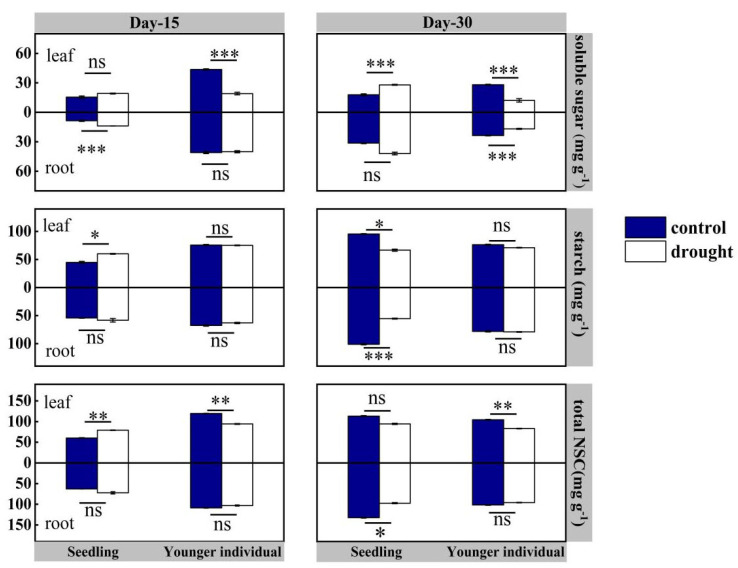
Leaf and root non-structural carbohydrates (NSC is soluble sugar plus starch) of seedlings and younger individuals of *H. ammodendron* at 15 d drought point (**left**) and 30 d drought point (**right**). The vertical bars represent the standard errors (*n* = 5). *, ** and *** represent significant difference at *p* < 0.05, *p* < 0.01 and *p* < 0.001, respectively. ns indicates no significant difference.

For seedlings, at the 15 d drought point, there was no significant difference in NSCs in roots between the control and drought group (*p* > 0.05), but leaf total NSC and starch contents of the drought group were higher than those of the control group, registering values of 79.14 vs. 60.28 mg·g^−1^ (+31.28%) and 60.00 vs. 44.84 mg·g^−1^ (+33.81%), respectively. At the 30 d drought point, both leaf and root total NSC and starch contents were lower than those of the control group, with leaf total content 94.48 vs. 113.17 mg·g^−1^ (−16.5%) and root total content 97.30 vs. 123.40 mg·g^−1^ (−21.2%), respectively (Figure 6).

For younger individuals, at both the 15 d and 30 d drought points, the leaf soluble sugars content of the drought group was significantly lower than that of the control group (−52.5% and −65.9%, respectively), while no significant difference in starch content was observed. Additionally, a significant reduction in leaf total NSC content was observed at 15 d and 30 d drought points (−21.0% and −20.3%, respectively). However, no significant difference in root NSC content was observed between the drought and control groups, except the lower soluble sugars content of the drought group at the 30 d drought point (−28.7%) (Figure 6).

## 3. Materials and Methods

### 3.1. Plant Material and Experimental Design

The experiment was conducted at Fukang Station of Desert Ecology, Chinese Academy of Sciences, in the southern Gurbantunggut Desert (44°13′ N, 87°33′ E, 475 m altitude, mean annual temperature 6.6 °C, mean annual precipitation 100~150 mm). We selected *Haloxylon ammodendron* for our research as it is a dominant species in this area. We investigated how developmental stages, specifically, the seedling and younger individual stages, of *H. ammodendron* influence physiological responses to continuous drought. At the beginning of the experiment, *H. ammodendron* seeds and one-year-old individuals were cultivated in pots (each pot with one individual) filled with 9.5 kg dry sandy soil from the southern Gurbantunggut Desert in March 2022. The seedlings and younger individuals were grown for about three months before imposing drought. We randomly assigned 80 individuals (40 seedlings and 40 younger individuals) to two groups: a well-watered control group and a drought group, with 20 of each type in both groups The control groups were watered normally (2 L water every three days), while drought groups underwent water restriction starting from 1 June. The leaf photosynthetic traits were measured at 15-day (15 d) and 30-day (30 d) points after drought treatments; accompanying this, twigs and roots were collected to measure water potential and non-structural carbon contents *(n* = 10 per treatment of seedlings and younger individuals).

### 3.2. Leaf Photosynthetic Traits

Photosynthetic traits were measured using a LI-6800 portable open gas exchange system (LI-COR, Lincoln, NE, USA) on twigs (leaves of *H. ammodendron* degenerate into twigs) of five individuals per treatment during each measurement period. Light-saturated net photosynthetic rates were measured between 9:00 and 13:00 h in a standard leaf chamber (6 cm^2^) equipped with a light source at PPFD of 1500 μmol m^−2^, an ambient CO_2_ concentration of 400 μmol mol^−1^ and a flow rate of 500 m s^−1^. The dark respiration rates were measured between 24:00 and 1:00 h after sufficient acclimation with no light, an ambient CO_2_ concentration of 400 μmol mol^−1^ and a flow rate of 500 m s^−1^. Following these measurements, the diameter and length of the twigs within the chamber were measured to calculate the net light-saturated photosynthetic rate per area (A_n_), dark respiration rate per area (R) and stomatal conductance (g_s_). Instantaneous intrinsic water use efficiency (iWUE) was calculated as the ratio of net photosynthetic rate to stomatal conductance (A_n_/g_s_). In addition, chlorophyll fluorescence measurements were performed using a LI-6800 Multi-Phase Flash Fluorometer (LI-COR, Lincoln, NE, USA). Measurements were performed using 250 ms duration multi-phase flashes of 10,000 μmol photons m^−2^ s^−1^ intensity and 250 kHz flash modulation rate. To ensure that plants were adequately dark-adapted, we determined the potential maximum photochemical rate of photosystem II (Fv/Fm) between 24:00 and 1:00 h.

### 3.3. Water Status

Five healthy twigs were cut per treatment before sunrise (5:00–6:00 h) to measure predawn water potential (Ψ_PD_) using a Scholander pressure chamber (PMS Instrument Company, Corvallis, OR, USA). Another five twigs were chosen for the midday water potential (Ψ_MD_) measurements, which were taken between 12:00 and 1:00 h. Five healthy twigs were cut from 3~5 individuals per treatment in a container full of ice to reduce water loss. The fresh weight of the twigs (W_fresh_) was measured as soon as they reached the laboratory; after measuring the fresh weights, the twigs were soaked in pure water under shade for over 24 h to obtain the water-saturated weight (W_sat_). The twigs were oven-dried at 75 °C until they reached a constant weight to obtain the dry weight (W_dry_). Leaf dry mass content (LDMC) and relative water content (RWC) were calculated as follows:

$$LDMC=\frac{W_{dry}}{W_{sat}}$$

$$RWC=\frac{\left(W_{fresh}-W_{dry}\right)}{\left(W_{sat}-W_{dry}\right)}$$

### 3.4. Non-Structural Carbohydrates

The low-molecular-weight sugars (glucose, fructose and sucrose), starch and total NSC contents (starch plus low-molecular-weight sugars) were measured [59]. The twigs and roots were collected and oven-dried for 72 h at 75 °C to avoid enzymatic activity. Then, the dry samples were ground to a powder. Soluble sugar content reagent boxes (BC0030, Solarbio Science & Technology, Beijing, China) and starch content reagent boxes (BC0700, Solarbio Science & Technology, Beijing, China) were used to determine the soluble sugars and starch contents from their solutions, respectively. The free glucose concentration was determined photometrically using a microplate photometer based on the absorbance of the sample solution at 620 nm according to the absorbance of the standard glucose reference solution. Starch content was determined by extracting the glucose content of sample solutions following the hydrolysis with sulfuric acid. Total NSC content was defined as starch plus soluble sugars contents, and all NSC values are presented as unit dry mass.

### 3.5. Data Analysis

A three-way repeated-measures analysis of variance (ANOVA) was used to test the effects of drought and developmental stage on gas exchange, water status and NSC content. Drought treatment, developmental stage and their interactions were analyzed as between-subjects factors, and treatment time, drought treatment, developmental stage and their interactions were analyzed as within-subjects factors. The values of some variables were transformed to meet the assumptions of normality and homogeneity. Then, differences in each variable between drought treatment and developmental stage were tested for each treatment time using a two-way ANOVA. Finally, a *t* test was used to analyze the effects of drought at each treatment time for developmental stage and drought treatment. All analyses were performed using R version 4.2.2 [60].

## 4. Discussion

This study reveals the different physiological strategies and gas exchange processes of *H. ammodendron* seedlings and younger individuals when responding to an extended duration of drought. For seedlings, the stomatal conductance decreased at the 15 d drought point and increased at the 30 d drought point (Figure 2). This result is consistent with many previous studies, where the photosynthetic rate under drought stress was significantly decreased due to stomatal closure to reduce transpiration [14]. Coupled with photosynthetic rate, stomatal conductance substantially determines iWUE, which sufficiently reflects water use strategies in plant tissue [61]. Compared with that of the control group, the iWUE of seedlings significantly decreased under drought stress, but this was not observed in younger individuals. The results suggest that seedlings close stomata partly or entirely to reduce water losses from the leaf, which may result in a further distance from optimizing water use efficiency [62,63]. A larger standard deviation in gs for seedlings at the 30 d drought point suggests a precarious ability to regulate stomatal conductance, which further suggests that the developmental stage is an important factor influencing drought response strategies and further influences survival after drought events [19]. Our findings revealed that *H. ammodendron* utilizes conservative stomatal regulation strategies, presenting positive stomatal regulation at the 15 d drought point. Similar to in previous studies, the stomatal conductance of younger individuals significantly decreased under drought stress to reduce water losses [30,31]. However, the g_s_ of seedlings decreased at the 15 d drought point but increased at the 30 d drought point. We speculated that the extended drought duration may lead to stomatal regular failure. According to the simultaneous decline in Fv/Fm at the 30 d drought point, extreme drought stress may induce a stronger photosynthetic inhibition of PSII and a further decrease in photosynthetic rate [62]. Due to the combined large reduction of water status with a significant reduction of Fv/Fm at the 30 d drought point, we considered that the severe injury of PSII may result in hydraulic failure because the reduction in hydraulic traits can inhibit the biosynthesis of chloroplast proteins [64]. Our result that the g_s_ reduced earlier than leaf water potential indicates that plants can close stomata to avoid hydraulic failure in response to little soil available water [63]. We inferred that the photosynthetic rates of seedlings were constrained by stomatal closure at the 15 d drought point and then constrained by biochemical inhibition at the 30 d drought point.

NSCs present multiple cofunctions in plant tissues, including growth, storage, osmotic regulation of hydraulically vulnerable leaves and recovery post drought [42,44]. At the 15 d drought point, there was no significant reduction in NSCs except in the leaf total NSC of younger individuals caused mainly by the reduction in soluble sugars. This result supports the point that carbon starvation takes time because plant carbon reserves appear resilient to extreme disturbance in the short term [38]. The earlier increase and later decrease in the total NSC content of seedlings demonstrated the time-logged impact of drought stress on carbohydrate metabolism [53]. Previous studies reported that stored carbohydrates can keep tissues alive for an extended drought period even with a reduction of photosynthetic rate [65,66]. Therefore, there is little possibility that *H. ammodendron* would experience carbon starvation under drought stress. Starch and soluble sugars take different roles in plant tissues. Starch is the main storage resource that can be mobilized to support growth and other functions [44,46]. In contrast, soluble sugars play an important role in osmotic regulation [67]. For seedlings under drought stress, starch may be hydrolyzed to increase soluble sugars concentration, which can increase osmotic potentials in the leaf [44,67]. On the contrary, younger individuals prefer to reduce NSCs in leaves as they can reduce carbon loss by foliage loss [68]. The stable root NSC content of younger individuals can maintain alive roots, which are an important foundation of regrowth in the absence of drought [47,69].

According to our result, we confirm that seedlings choose to take a risky strategy to try to maintain carbon assimilation rather than invest in roots to obtain the little water, which may need more investment. By the way, we noticed the strong susceptibility of NSC content to environmental factors in all organs. Previous research on mature *H. ammondrendon* in situ revealed a significant increase in starch content and decrease in soluble sugars content compared with the control groups [56]; in our study, no significant decrease in starch content was observed in *H. ammondrendon* individuals, except seedlings during severe drought stress. Previous research has revealed the gene expression of a drought-tolerant cultivate of flax at different developmental stages responding to drought stress [70,71]. We appeal for further study to reveal the potential mechanism underlying the multiple changes of NSCs among organs to enhance the understanding of product regulation at different ages of plants under drought stress.

## 5. Conclusions

**Figure 7:**
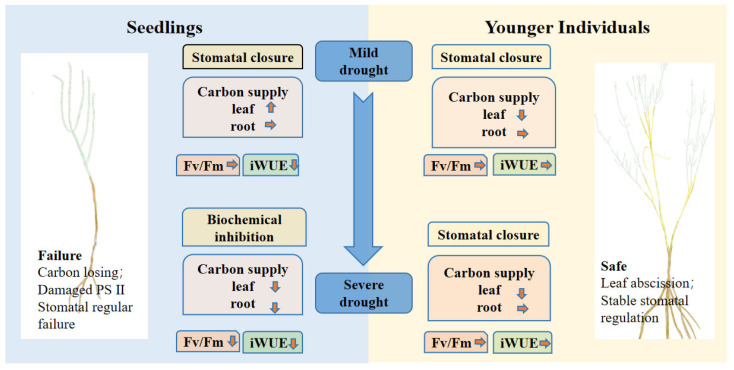
Conceptual diagram of seedlings’ and younger individuals’ responses to drought stress. The direction of the little arrows indicate the changes of NSC contents of the drought group compared with the control group, the up arrow represents an increase, the down arrow represents a decrease and the right arrow represents no change.

Our results suggest that the effects of a plant’s developmental stage on stomatal conductance strongly depend on drought duration, which indicates the limitation of the regular ability of seedlings under severe drought stress. According to the physiological response of seedlings to drought, we affirm that seedlings prefer risky survival strategies because the seedlings during severe drought periods were characterized by dysfunctional physiological regulation (Figure 7). More importantly, re-opening stomata under extreme water conditions may result in high hydraulic failure risk. Considering the changes of NSCs under different drought duration, NSC content of seedlings experienced firstly an increase and, following, a decrease over the duration of drought due to biochemical inhibition. Combining leaf photosynthetic traits and NSC contents, we can conclude that seedlings have limited ability when responding to reduced soil moisture compared to younger individuals under a prolonged drought period, suggesting that younger individuals can be widely used in vegetation restoration of the desert ecosystem.

## Figures and Tables

**Table 1:** Summary of three-way repeated-measures ANOVA *p*-values for treatment effects on plant water status, leaf gas exchange and NSC content in *H. ammodendron*. A_n_, net photosynthesis rate (μmol m^−2^ s^−1^); R, dark respiration rate (μmol m^−2^ s^−1^); g_s_, stomatal conductance (mol m^−2^ s^−1^); iWUE, intrinsic water efficiency (mmol m^−2^ s^−1^); Fv/Fm, potential maximum photochemical rate; LDMC, leaf dry mass content (g g^−1^); RWC, relative water content (g g^−1^); L-sugar, leaf soluble sugars content (mg g^−1^); L-starch, leaf starch content (mg g^−1^); L-NSC, leaf total non-structural carbohydrates (mg g^−1^); R-sugar, root soluble sugars content (mg g^−1^); R-starch, root starch content (mg g^−1^); R-NSC, root total non-structural carbohydrates (mg g^−1^). *p*-values in bold are significant (*α* = 0.05).

Source of Variation	A_n_ μ mol m^−2^ s^−1^	R μ mol m^−2^ s^−1^	g_s_ μ mol m^−2^ s^−1^	iWUE μ mol m^−2^ s^−1^	Fv/Fm	LDMC g g^−1^	RWC g g^−1^	L-Sugar mg g^−1^	L-Starch mg g^−1^	L-NSC mg g^−1^	R-Sugar mg g^−1^	R-Starch mg g^−1^	R-NSC mg g^−1^
Within subject													
Treatment duration	**<0.001**	**<0.001**	**0.017**	**<0.001**	**0.006**	**<0.001**	**<0.001**	**0.021**	**0.004**	**0.010**	0.629	**<0.001**	**<0.001**
Treatment duration and development stage	**0.030**	**<0.001**	**0.017**	0.164	**0.005**	**<0.001**	**<0.001**	**<0.001**	**<0.001**	**<0.001**	**<0.001**	0.251	**0.001**
Treatment duration and drought treatment	0.218	0.089	**0.016**	0.624	**0.002**	**<0.001**	**<0.001**	**0.028**	**0.010**	**0.043**	0.501	**0.0152**	**0.013**
Treatment duration and drought treatment and development stage	0.392	0.769	**0.008**	0.522	**0.019**	**<0.001**	**0.003**	0.777	**0.009**	**0.008**	**0.009**	**0.006**	**0.023**
Between subject													
Drought treatment	**<0.001**	**0.046**	0.857	**<0.001**	**<0.001**	**<0.00**1	**<0.001**	**<0.001**	**0.078**	**<0.001**	0.704	**0.005**	**0.015**
Developing stage	**0.024**	**<0.001**	**0.034**	**<0.001**	**0.002**	**0.035**	**<0.001**	**<0.001**	**0.007**	**<0.001**	**0.017**	0.139	**0.021**
Drought treatment and development stage	**0.014**	0.157	**0.015**	**<0.001**	**0.007**	**0.002**	**0.005**	**<0.001**	0.461	**<0.001**	**0.029**	**0.012**	0.177

## Data Availability

Data will be made available on request.
